# Three Cooperative Extension initiatives funded to address Michigan's opioid crisis

**DOI:** 10.3389/fpubh.2022.921919

**Published:** 2022-09-20

**Authors:** Cheryl L. Eschbach, Dawn A. Contreras, Lauren E. Kennedy

**Affiliations:** Health and Nutrition Institute, Michigan State University Extension, East Lansing, MI, United States

**Keywords:** community-based education, health extension, healthcare referrals, opioid misuse prevention, rural

## Abstract

People living with opioid use disorder and those experiencing other types of substance misuse are part of a public health crisis in the United States. Rates of opioid misuse, overdose, and opioid-related deaths within different subpopulations show where prevention efforts must focus. Through concerted efforts, aligned with common goals, a statewide community-based educational organization (Michigan State University Extension) has demonstrated ability to acquire multi-year funding from varied sources of state and federal funds that has produced robust support for statewide projects and collaborations. Researchers, educators, public health program managers, and other practitioners can benefit from learning how three funded initiatives in one state resulted in improved awareness and access for individuals and healthcare organizations. By sharing our implementation of health educational programs and presentations, other states' can adopt these evidence-based strategies for similar outreach. Cooperative Extension in Michigan delivers program series and one-time education to the public on the self-management of chronic conditions and pain, mindfulness for stress reduction, anger management, and opioid misuse prevention, treatment, and recovery. These evidence- and research-based health programs implemented by Extension staff teach participants common aspects of prevention such as self-management care, communication skills, self-efficacy, and goal setting or personal health action plans. Education aims to reduce dependency on opioids, prevent opioid misuse and share non-pharmacological solutions to pain management for those living with chronic conditions or at risk for developing dependence. The funded initiatives targeted rural residents, older adults, health care providers, and people living with chronic pain who may have access to prescription opioids. In addition to direct education, projects supported local communities with the development of coalitions, including the training of community partners to become program facilitators thereby increasing community capacity for prevention programs, and through the creation of patient referrals from healthcare settings to community-based education. In rural areas, Cooperative Extension plays a crucial role in connecting community resources to address healthy aging, and chronic disease or chronic pain self-management education. Community partners engaged in public health education and promotion, and healthcare providers alike may not be aware that Cooperative Extension plays a vital role in providing community-based health education.

## Introduction

Substance misuse and the resulting harm is a public health crisis in the United States. State level trends of opioid misuse, overdose, and opioid-related deaths within different subpopulations show where prevention efforts should focus. Prevention efforts are coordinated and intentional activities that seek to reduce harm to individuals and support the promotion of healthy behaviors and outcomes (1). Creating strategic prevention efforts means using assessments, capacity building, planning and program implementation, as well as evaluation to conduct culturally sensitive projects with sustainable principles (1). Community-based education is one way individuals learn strategies (i.e., knowledge and behaviors) for improving their health, and when these educational outreach efforts work in partnership with others in the community, it can improve health care delivery and ultimately access to healthcare for all (2).

This article documents years of sustained statewide efforts of a community-based educational organization, operating with various funding sources to implement concurrent initiatives. Need for educational programming to address the public health issue of opioid misuse is presented with a description of one state's implementation strategies. Evidence shows how a community-based educational organization like Cooperative Extension can assist health care professionals in increasing awareness of health programs available to patients through partnerships and referrals. Implications for practice are shared so that other states may recognize the value of Cooperative Extension as an effective outreach partner for prevention strategies (2) and so that other Cooperative Extension systems nationwide can adapt similar strategies.

### Opioid misuse, overdose, and opioid-related deaths

Over 10 million people misuse prescription opioids each year in the United States (3). For prevention efforts, community practitioners are encouraged to use existing state and local data to prioritize problems for outreach, such as looking at magnitude, severity, and trends (1). Michigan mirrors the United States in problems of misuse but is above the national average for drug overdose deaths. In 2018, Michigan was ranked 12^th^ in the nation for opioid-involved overdose deaths per 100,000 persons (4). During this time, Michigan also ranked 10^th^ nationally for per capita prescribing rates (4). Michigan's higher rates of prescribed opioids in circulation contributed to higher rates of opioid-related overdoses and deaths, highlighting a need for prevention efforts to address the public health crisis of opioid misuse. Opioids are prescribed for valid medical reasons such as the management of chronic pain. Yet, opioids are highly addictive and can quickly lead to misuse and Opioid Use Disoder (OUD), particularly for those already made socially vulnerable due to their race, ethnicity, gender, age, or other marginalized identity. Misuse is defined as taking a prescribed opioid medication but in higher quantities, higher frequencies, or for a longer than prescribed (5). Misuse also includes taking someone else's prescription or using an opioid to feel high (5). The COVID-19 pandemic has intensified the impact of the opioid crisis nationally, as well as in Michigan, with data showing overdose deaths increased and were likely undercounted in 2020 (6). Disturbing racial disparities in opioid-related overdose deaths show the age-adjusted opioid overdose mortality rate fell by 16.9 percent for white residents but rose by 0.7 percent for Black residents (6). Impacts of the COVID-19 pandemic on the opioid public health crisis are still unfolding, yet recent data are showing opioid overdose death rates (nationally and in Michigan) have risen since 2020. Forced isolation, reduced and limited access to health services, and self-medicating behaviors have influenced the sharp increase in opioid overdose rates (7). Data from 12 months prior to April 2021 show increases in overdose and death rates attributable to illicitly manufactured synthetic opioids like fentanyl showing up in the opioids supply and use of psychostimulants such as methamphetamine (7).

### Education helps prevent opioid misuse

Data obtained from 11.5 million Americans (8) indicates that people most report misusing opioids to relieve physical pain, to feel good or get high, to relax or relieve tension, and to help with sleep or help with feelings or emotions. Individuals can learn and practice specific skills they can use to address their physical pain and stress. Due to the isolation of residents in rural areas and the lack of access to healthcare providers, (9) telehealth and distance learning strategies are also needed to reach underserved, rural residents. Primary care providers and public health officials often do not have adequate time and resources for outreach so they can teach patients knowledge or behaviors to reduce symptoms or promote health literacy and healthier lifestyles (10). Previous efforts to help people manage obesity found engagement tools were needed to form clinical and community connections (10) where physicians make informal referrals to local resources. Health education strategies support rural populations with evidence-based prevention programs in ways that are convenient, inexpensive or offered at no cost, and easily accessible (2).

## Context

This community case study describes implementation of several evidence-based strategies and funded initiatives to address the opioid crisis by a statewide educational health service organization. While many organizations provide statewide, community-based educational programming for adults on such health issues as diabetes prevention (11) or nutrition education (12), few have delivered wide-spread prevention education on opioid misuse. This novel case study shares statewide approaches to address the opioid crisis with prevention education and other public health management strategies from a period between 2018 and 2022.

Collectively, strategies from three funded initiatives include direct health education reaching individuals with community-based programming, coalition building through organizational partnerships, leader trainings for community members to become trained facilitators of evidence-based prevention programs, and the establishment of referral pathways between healthcare and health educational programs for patients. This comprehensive, multi-level, and multi-year approach to opioid misuse prevention provides evidence to other practitioners about the effective approaches in planning and implementing preventative initiatives. Funding, staffing, program delivery, and outcomes associated with prevention strategies are highlighted so that others can adopt similar initiatives to achieve similar outcomes.

### Focus on older adults, adults living with disabilities, and rural residents

In Michigan, direct education efforts for preventing opioid misuse and overdose rates or death have focused on subpopulations such as older adults, adults living with physical disabilities or chronic health conditions, and rural residents. Opioid prescriptions are often given to older adults and adults living with disabilities because they more commonly suffer from chronic pain and chronic conditions (13). Prescription opioids, combined with physical and emotional pain experienced by older adults and adults with disabilities or chronic conditions, can lead to behavioral health issues, such as substance use disorder (SUD) (13). Risk factors that make older adults and adults with chronic conditions particularly vulnerable to drug overdoses include combining opioids with certain other drugs and/or alcohol, medical conditions that affect drug absorption, and an age greater than 65 years (14). Research shows adults with physical disabilities have an almost three times higher risk of death from opioid overdose than adults without a disability (14). In 2022, one in every four Michigan residents has a documented disability (e.g., mobility, cognition, hearing) (15).

### Equity issues for black residents and COVID-19 impacts

Whereas, opioid poisoning mortality rates among white residents decreased slightly between 2017 and 2018, rates among Black residents increased by nearly 20 percent (16). Inequitable access to appropriate treatments for pain management, opioid use disorder, or other chronic health conditions (17) is further compounded by a history of framing addiction as a criminal issue for communities of color, rather than a public health or medical issue, requiring treatment and care (18). Moreover, people of color tend to make up a smaller percentage of the population in rural areas, including in Michigan (19). As a result, these individuals are often overlooked in prevention or treatment efforts, further marginalizing them. With the understanding that racism is found at multiple levels (e.g., interpersonal, institutional) within systems (e.g., health care), even when opioid use disorder and misuse are framed more accurately as a medical or public health issue, the likelihood of experiencing differential access or care remains high (20, 21). Prevention efforts are, therefore, limited by the extent to which racism is considered as a contributing factor to the opioid crisis (22). The COVID-19 pandemic presents additional challenges, increasing the risk for initiating opioid misuse, (23) as well as interfering with care for those undergoing treatment for OUD (24). Michigan data shows increases in opioid-related deaths that may be attributable to the pandemic (23). While the relationship between opioid misuse and COVID-19 continues to emerge, both public health crises have disproportionate effects on Black Americans, warranting prevention approaches that take equity into account (2, 25).

### Michigan's cooperative extension health programs as prevention strategies

Cooperative Extension is an outreach unit of land-grant universities that takes research-based information to the public through various delivery mechanisms (e.g., direct education, awareness campaigns, systems changes) (2). Cooperative Extension has community-based educators who work with researchers to develop and implement research-informed and evidence-based programming and resources. In addition to serving agricultural, entrepreneurial, and youth development (4-H) audiences, Cooperative Extension is also a community-based health education organization that responds to shifts in health priorities and emerging issues (2).

Our state's Cooperative Extension organization is Michigan State University (MSU) Extension. MSU Extension's health programs teach participants common aspects of prevention such as self-management strategies, communication skills, self-efficacy, and goal setting or personal health action plans. MSU Extension focuses on staff development with dedication to onboarding best practices (e.g., mentorships, peer teaching observations) and professional training. MSU Extension health educators hold graduate degrees, select programs from a statewide list based on community needs, and serve communities as trained facilitators in evidence- and research-based programs. This organizational structure results in the implementation of community-based health programs, locally managed by staff dedicated to geographic programming districts, which creates statewide coverage. Educators implement programs in-person and are trained to teach content online. Training several staff in the same program produces teams of co-facilitators who can work together to plan in-person and online implementation of health programs.

MSU Extension staff plan and implement prevention classes to thousands of residents each year through multi-session, evidence-based prevention courses and through delivery of one-time presentations. One-time presentations (called Session Zero) are a strategy to recruit participants into longer series. Session Zero prevention classes include information on alternatives to pain management medication and ways to prevent opioid misuse (e.g., knowing signs and symptoms, keeping medications safely stored). Telehealth goals are achieved with online programming using Zoom technology, which includes options for telephone and mobile phone app connections. In Michigan, the history and purpose of the Cooperative Extension organization coupled with the structure and function of educator teams organized around key health outcomes has resulted in three projects with concurrent state and national funding.

### Working with healthcare professionals to increase awareness of health programs and referrals to education from primary care

To assist organizational goals of effective outreach to new healthcare audiences, MSU Extension collaborated with the American Medical Association to survey physicians in state (26). Results suggested a need to increase awareness of MSU Extension programs. Overall, physicians have positive attitudes about how educational programs help patients but shared perceived barriers to making referrals because of their limited knowledge about programs (26). Results of the survey informed strategies implemented in Michigan to address the opioid crisis with education.

One way to lessen the burden on healthcare providers of developing a referral system is to provide a tool and process they can use to refer patients to community-based programs. To achieve this goal, MSU Extension first developed the *Rx for Health Referral Toolkit*, which contained a paper referral pad and instructional guides, to educate providers and create a means for referrals into MSU Extension health programs. The referral pad instructed patients to contact their local Cooperative Extension office by phone or by visiting the office for program information and enrollment. Implementation of the Rx for Health toolkit was limited by providers' reluctance to use a paper referral pad, (27) justifying the future need for an electronic referral system. Funding was needed for MSU Extension to pursue an electronic referral system for patient referrals into health programs.

## Details of program implementation

MSU Extension implements different health programs as direct education to address opioid misuse prevention strategies or intervention. These programs (offered in-person and online) are characterized as self-management, mindfulness teaching with intent on stress reduction, and anger management.

### Research- and evidence-based health education to address the opioid crisis

The Chronic Pain Self-Management Program (CPSMP) and Chronic Disease Self-Management Program (CDSMP) are evidence-based program series with documented improved outcomes related to pain, fatigue, medication adherence, quality of life, sleep, health distress and communication with healthcare providers, (28) including among patients with depression and serious mental illness (29, 30). CDSMP and CPSMP are evidence-based programs listed on the *Centers for Medicare and Medicaid Services' Evaluation of Community-based Wellness and Prevention Programs*. CDSMP and CPSMP are 2$\frac{1}{2}$-h sessions taught weekly for 6 weeks. MSU Extension has implemented CDSMP and CPSMP since 2009 and has four master trainers. In 2021, MSU Extension began implementing a PATH toolkit option for participants, which relies on mailing print materials to them and connecting rural residents by telephone. Stress Less with Mindfulness (SLM) is a program series that introduces individuals to a variety of mindfulness techniques and is taught in five, weekly, 1-h lessons. Documented program outcomes include increased personal self-awareness of stress symptoms and use of mindful breathing and mindful movement to calm the body and mind. SLM is a research-based and practice-tested program (31). The RELAX: Alternatives to Anger program helps participants manage anger and stress by developing communication and problem-solving skills needed for healthy relationships (32). Through a series of four lessons, participants learn non-pharmacological approaches to manage psychological pain and foster healthy, supportive relationships. Key elements of RELAX include learning what anger is, what triggers anger, calming down and destressing methods, problem solving, communication skills and forgiveness (32).

Educational approaches to prevent opioid use and misuse within Michigan were possible because of external funding awarded to Cooperative Extension. During times when state and federal funding allocations for Cooperative Extension has remained stagnant, competitive awards have created new opportunities. Initiatives were multi-year projects working concurrently with funding from the United States Department of Agriculture's National Institute for Food and Agriculture (USDA NIFA), the Substance Abuse and Mental Health Services Administration (SAMHSA), and Michigan's Department of Health and Human Services (MDHHS). Since 2018, MSU Extension has leveraged these funding sources to rapidly expand the organization into community behavioral health by adding capacity for Extension to engage in funded initiatives and build upon best practices discovered under each funded project. Each of the projects had a direct education component. MSU Extension core programming in health education led us to start these initiatives on opioid misuse prevention and seek funding to grow or scale up this new area. Local Extension Educators had monthly meetings for training to discuss best practices on marketing, implementation, evaluation, and progress updates. Table 1 shows the characteristics of health programs participants and outreach audiences by the three funding sources. Demographic data of 2,937 participants is available, however, it is not inclusive of all participants who completed programming due to the voluntary design of the evaluations and a measurement focus on rural audience reach.

**Table 1:** Characteristics of rural health program participants and outreach audiences for opioid misuse prevention education by funding.

**Funding source for initiatives (years funded)**	**State opioid response (SOR) state department of health and human services (2018–2021)**	**SAMHSA rural opioid technical assistance (MiSUPER) (2019–2022)**	**USDA NIFA rural health and safety education (RHSE) (2020–2022)**
Educational health programs implemented to address opioid misuse prevention	Self-management; mindfulness, stress management series (*n* = 621)	One-time presentations (*n* = 408)	Self-management; mindfulness, stress management; anger management series (*n* = 1,010)
**Characteristics**	*n* (%)	*n* (%)	*n* (%)
Gender			
Women	513 (84%)	363 (90%)	405 (86%)
Men	98 (16%)	38 (10%)	67 (14%)
Race/ethnicity			
Native American	7 (1%)	21 (5%)	22 (5%)
Asian	6 (1%)	4 (<1%)	5 (1%)
Black/African American	47 (8%)	4 (<1%)	23 (5%)
White/ Caucasian	542 (90%)	373 (91%)	421 (89%)
Hispanic/Latinx	7 (1%)	14 (3%)	25 (5%)
Age			
55 years and older	621 (100%)	96 (24%)	174 (37%)
Education			
High school diploma	16 (10%)	15 (4%)	108 (24%)
Some college	23 (15%)	33 (8%)	82 (19%)
College degree	117 (75%)	359 (88%)	254 (57%)
Lives alone	142 (29%)	38 (11%)	55 (14%)
Lives with others	354 (71%)	322 (89%)	403 (86%)

Demographics are for rural participants determined by self-reported residence ZIP code. MiSUPER project reached 1,458 participants in a 2-year timeframe and 408 evaluation surveys were returned voluntarily with self-reported demographic information from participants. Race and ethnicity were not mutually exclusive and participants could report all responses that apply.

### Funded initiative 1: State opioid response 2018–2021

In May 2018, the state health and human services department contacted MSU Extension to provide community-based substance use disorder prevention education to Michigan's adults, aged 55 and older. Of particular focus were older adults living with physical disabilities that caused chronic pain or a need for self-management and/or caregiving for assistance in daily activities. This included adults who may have a valid prescription for opioids to manage their pain symptoms but who could benefit from education on the signs and factors of misuse or addiction. This effort was funded for 3 years as part of Michigan's State Opioid Response (SOR) and SOR Supplemental grants with the state's primary funding from SAMHSA. This initiative reached over 3,100 unduplicated older adults with education related to non-pharmaceutical approaches to pain management.

### Funded initiative 2: Michigan substance use prevention, education and recovery 2019–2022

In October 2019, MSU Extension started an effort called MiSUPER (Michigan Substance Use Prevention, Education and Recovery). Funded by SAMHSA's Rural Opioid Technical Assistance (ROTA) grant program, MiSUPER is a collaborative project with MSU Extension, the MSU College of Human Medicine Department of Family Medicine, and the Health Department of Northwest Michigan. The goal of MiSUPER was to increase awareness of opioid misuse in rural communities and share prevention, substance use disorder treatment options, and recovery support education. Both community members and healthcare professionals attended trainings designed with their interests in mind, during which they learned to recognize signs of misuse and options for professional treatments and support for those in recovery. The project surpassed outreach goals in implementing virtual online trainings on opioid misuse prevention, treatment, and recovery. MiSUPER hosted 45 events in 2 years, reaching 1,458 participants with education. Demographics are available for 408 individuals living in rural areas who responded voluntarily to evaluation surveys (Table 1). The project built upon MSU Extension's other opioid misuse prevention educational initiative (SOR) and added new behavioral health educators to the organization. For the first time, MSU Extension expanded its focus beyond prevention education to include treatment options and recovery community supports in opioid-related education and materials.

### Funded initiative 3: USDA NIFA rural health and safety education 2020–2022

A third funding source for MSU Extension to address opioid misuse prevention statewide was received in September 2020 from USDA NIFA's Rural Health and Safety Education (RHSE) funding program. The project has a multi-level, four-aim approach to respond to the opioid crisis in rural Michigan. The first aim works with Communities that Care (CTC) coalitions in the rural counties of Michigan's Upper Peninsula to build awareness, make collaborative plans to implement evidence-based opioid misuse prevention programs, and explore ways MSU Extension can work with local coalitions to address racism that contributes to inequitable opioid misuse outcomes. The second aim cross-trains recovery coaches and coalition members to facilitate evidence-based programs locally. The third aim uses marketing and technology strategies to equip physicians and mid-level healthcare providers (including care managers in clinical practices) across rural counties in Michigan to promote patient referrals into prevention programs.

The USDA NIFA project funded a subscription to *Referrals Plus* software, creating an electronic solution to a previously identified need and supported hiring a half-time program referral coordinator. The fourth aim was direct education to implement evidence-based opioid misuse prevention education programs with residents in all of Michigan's rural counties to empower them to use non-pharmacological approaches in pain management. Based on past successes with the MiSUPER project, MSU Extension again partnered with the MSU College of Human Medicine Department of Family Medicine on this comprehensive approach to leverage content specialty.

### Program evaluation plan and tracking strategy effectiveness

This project tracked progress on the following long-term outcomes: increasing awareness of public health educational prevention programs in rural communities, increasing awareness and reach of programs *via* increased referrals and enrollment, engaging rural areas by training instructors, establishing connections between healthcare providers and MSU Extension's research- and evidence-based health programs, increasing knowledge, skills and healthy behaviors related to preventing or reducing opioid misuse and increasing awareness of non-pharmaceutical approaches for pain and chronic disease symptom management. A program logic model (Table 2) shows collective inputs, activities, outputs, and outcomes across the three funded initiatives.

**Table 2:** Logic model for three cooperative extension initiatives funded to address Michigan's opioid crisis.

**Inputs**	**Activities**	**Outputs participation**	**Outputs activities**	**Outcomes**
•MSU Extension staff: Specialists and Educators •MSU Department of Family Medicine Faculty •MSU College of Human Medicine Family Residency Program •Coalitions •State and federal funding (i.e., SAMHSA ROTA, USDA NIFA RHSE) •Healthcare providers and allied health professionals •Program and training materials including MSU Extension developed curricula for mindfulness/stress reduction and anger management (e.g. RELAX and Stress Less with Mindfulness), including master instructor trainings for both programs •Existing staff trained in chronic disease self-management program (CDSMP) and chronic pain CDSMP, including three master trainers and license held by MSU to provide leader trainings •Project websites hosted by MSU	Coordinate prevention work with community coalitions (*via* meetings) Train-the-trainer sessions with teach back assessments, background checks, and observations Develop healthcare referral pathways by updating Extension webpages with available program series and creating an electronic referral mechanism for providers Provide printed materials and QR code for referral process to health care partners Plan, enroll, implement, evaluate community-based prevention education programs	Engagement with rural coalitions •Programs marketed as co-branded and jointly planned •Cross-referrals into prevention programs •Recovery coaches/coalition members enroll for instructor training and stay engaged Number of providers making referrals Program participants - Adults aged 18+ who enroll and attend, including demographics Attendance by series and by session	Established model for Health Extension, including telehealth strategies, from collaboration with the family medicine residency network at MSU College of Human Medicine campuses and clinics Tested effective models for web-based/ electronic healthcare referral pathways to community education Created models for Extension's collaboration with coalitions in rural areas Decreased opioid misuse prevalence and associated stigma Improved support of community peers (e.g., recovery coaches, coalition members) Increased health literacy of rural residents *via* access to information about health promotion activities, such as self-management programs Improved collaborative strategies among an Academic Health Center, Cooperative Extension, and local coalitions, leading to outcomes such as prevention and reduction of opioid misuse, including the promotion of alternatives to prescribed pain management	Increased awareness of community needs and public health educational prevention opportunities in rural communities Increased awareness and reach of programs *via* increased referrals and enrollment (number of program participants) Increased engagement in rural areas with trained cohort of instructors Established connections between health care providers and MSU Extension's research- and evidence-based programs Increased knowledge, skills, healthy behaviors related to preventing or reducing opioid misuse Increased awareness of non-pharmaceutical approaches to pain symptoms and chronic disease management

Situation: Rural Michigan has been impacted by the opioid crisis and would benefit greatly from multi-level, multisectoral community-based interventions. Although MSU Extension has demonstrated success partnering with healthcare providers to refer patients into evidence-based prevention programs, the mechanism had not yet been adapted for telehealth or distance education.

External factors include varying broadband access in rural areas; public health pandemic protocols and emergency plans activated; and unexpected changes in program resources or foundational funding. Assumptions for the logic model include cooperation among MSU Extension and MSU partners, that community coalitions and persons in recovery will be interested and participate in training; providers will be willing to attend trainings and will use an external online portal for program referrals; and addressing social and structural determinants of opioid misuse requires multi-level intervention with multi-sectoral collaboration.

Common activities include creating awareness between community coalitions and MSU Extension through meetings and surveys. MSU Extension provides train-the-trainer courses for community members, such as recovery coaches, to become trained to deliver and lead prevention and self-management programs in their communities. Marketing materials promote awareness and describe the health programs available to encourage patient referrals to community-based education. The USDA NIFA funds focused on telehealth have supported MSU Extension in setting up technology systems and marketing strategies to create various pathways to receive electronic patient referrals from primary care providers and family medicine clinics. Printed brochures highlighting available programs and how to sign up for free health classes were mailed to 1,140 family medicine and pain clinics as part of awareness outreach. An additional 357 clinics were followed-up with phone calls and individual emails. A lesson learned was that clinics were not interested in using an additional electronic referral system on the server to make referrals, they preferred ways to provide printed materials to providers and patients, to make verbal recommendations for the educational programs, and to have access to a website *via* QR code for the patient to follow-up with Extension about the available programs. In addition to this system change work, MSU Extension is still engaged in direct education outreach, much like the SOR project, by implementing and evaluating four research- and evidence-based health programs implemented statewide to participants (i.e., chronic disease self-management, chronic pain self-management, mindfulness and stress reduction, and anger management programs). Collection of outcome program evaluation data on direct education efforts is ongoing.

## Implications for practice

Community-based organizations that are accessible to state residents in every county provide a network to reach people with education. Cooperative Extension's national network, aligned with land-grant universities, has fiscal-management and staffing capacity to make meaningful impacts through effective health education programs. Educational efforts focused on prevention of opioid misuse and OUD need to be tailored to healthcare providers and community member audiences. The MSU College of Human Medicine and MSU Extension partnership in these funded efforts can serve as a model to promote health partnerships nationwide between Cooperative Extension within land-grant universities and academic health centers or community-based medical schools (33). Partnerships with Cooperative Extension in any state can contribute toward the goal and on-going work of reducing inequities in opioid misuse outcomes for state residents. Most people rely on their healthcare providers for information on healthy lifestyle behaviors, such as beginning or maintaining an exercise program (34). Likewise, physicians identify social needs as very important to address with patients and they express a need for connections to community resources (35). This suggests that healthcare professionals could benefit from more familiarity with available programs like those offered by Cooperative Extension.

Intentional programming with Black Americans is needed in future opioid misuse prevention efforts undertaken by Cooperative Extension. Our current funding sources from USDA NIFA limit reach to marginalized groups because of the focus on rural geography as the target for educational outreach. If we know people of color tend to be under-represented in rural areas, then our funding sources need to recognize value in going beyond ZIP codes or county targets to identity audiences, and to also include a focus on how structural racism contributes to differential access to prevention, treatment, and recovery supports. Within our case study, we noticed that marginalized audiences were underrepresented in our educational programming. One possible reason might have been a mistrust or fear of engaging with an organization that is associated with a university. To overcome this barrier, projects may need to partner with entities that have better social capital with under-represented groups, such as local champions within the group or venues with a trusted relationship with the group. Another way we sought to connect with under-reached minoritized groups was by working through and with coalitions. A primary step of working with coalitions in rural areas is determining if these coalitions are already addressing social determinants of health or considering racism in their work so that we could identify new opportunities to work together. Other organizations that have similar outreach projects might also find that coalitions are not at a state of readiness to engage in these issues. If this barrier occurs, it is important to pause on direct programming and build awareness together.

Another reason why certain groups might not have engaged is a possible stigma associated with the topic of substance abuse. To overcome this barrier, we used positive messaging (i.e., “misuse can happen to anyone”; “recovery is possible”) and stayed away from imagery like pills on our media and marketing. We also discussed OUD as a brain disease or chronic condition, instead of a personal choice or intentional behavior. In our community case study, we trained participants to use “person-first language,” so they could reduce stigma in how they talk about the public crisis with others. It was important in rural areas to not use fear or punitive-based tactics to encourage voluntary participation in community education. Instead, we found in our community case study that a positive approach was much more effective.

Additional barriers found in this case study were ones especially relevant to working in rural areas, including limited access to internet and equipment to participate in online learning. A technique we used to overcome this barrier was offering phone-based options for education. A second barrier to programming in rural areas is limited transportation for participants. Offering programming at residential settings and locations where participants routinely travel helped to overcome this barrier. Example programming sites included senior centers and residential drug treatment facilities.

## Discussion

All projects demonstrated the value of partnerships to community-based prevention work. Several partnerships were key in Cooperative Extension being competitive for funding and for successful project implementation. The strongest partnerships included: the state department of health and human services; the MSU College of Human Medicine; and existing community coalitions in the state. Building partnerships required meetings with other MSU faculty engaged in this academic content area and learning from and networking with other groups funded to do similar projects in the same coverage areas, including other universities and medical schools in the state. A common goal among partners was to prevent opioid misuse, overdose, and opioid-related death among Michigan's residents. While we had important common outcomes to work on with partners, engagement of partnerships was also a deliberate approach in the strategy and one we suggest to other practitioners.

Sustainability efforts look like securing funding from different agencies on the same public health topic with intention to scale up multi-level projects (e.g., individual direct education and health care system transformation). A key to sustainability is an organization's ability to retain core staff trained in evidence-based program delivery who can implement project objectives. Sustainability has also meant investing in electronic referral software, new staffing, and solid website development for program promotions, marketing, and program enrollments. Funding models are expected to change, such as the SAMHSA ROTA grants becoming regionalized technical centers instead of state-level projects. Sustainability in our state means being a partner on multi-state grant applications and creating new training contracts at the state department level for professional development to prevention and substance use staff. This change in funding model makes existing partnerships for Extension (e.g., College of Human Medicine) more important to have established. As an iteration of funding, states might consider a change to technical assistance, instead of a state-level project. Multi-state funding opportunities force teams to work together with fewer resources and requires awareness of each other's strengths in the same content area. ROTA funding allowed us to develop an infrastructure for future growth that included: a partnership with family medicine and a health department; a robust website and online presence for Extension's work in this content area; and standardized and evaluated for effectiveness community presentations that provided continuing education credits and professional development for individuals working in community services.

These three funded initiatives provided MSU Extension capacity building and growth in addressing behavioral health in Michigan and positive sustainable effects are expected for years. Strengthened relationships with healthcare providers, as well as increased familiarity with prevention programs and patient referral mechanisms, may lead to years of collaborations to address the opioid crisis or other public health issues. MSU Extension's strategies in these funded projects were to connect healthcare providers with information on community-based health programs to refer patients to education. The electronic menu of programs, patient referral forms and referrals pathways are embedded in MSU Extension's website and will be maintained as resources to broaden outreach to residents statewide into the future. Lessons learned from educating rural residents through telehealth and distance learning modalities are expected to increase future rural outreach effectiveness for years to come. In rural areas, Cooperative Extension plays a crucial role in connecting community resources to address chronic disease self-management education (36). Healthcare providers may not be aware that Cooperative Extension also plays a vital role in providing community-based health education.

Limitations discovered in recent efforts can be framed as opportunities to expand, scale up, or improve current programmatic efforts. Future efforts can provide education and resources for harm reduction approaches and treatment options (e.g., MOUD); partner with Black families, particularly Black rural families and other minoritized subpopulations; adopt an approach that includes a deeper level of engagement with community members, as well as work alongside people who use drugs, and those in recovery to strengthen outreach efforts. Future efforts could focus on documenting implementation strategies of these community-based projects funded from different sources working concurrently and overlay them with published, well-evidenced implementation strategies from the clinical literature (37, 38). This would provide a detailed implementation roadmap for other states' Cooperative Extension systems and their partners to adopt and integrate research- and evidence-based community behavioral health programming.

## Data availability statement

The original contributions presented in the study are included in the article/supplementary material, further inquiries can be directed to the corresponding author.

## Ethics statement

The studies involving human participants were reviewed and approved by the Institutional Review Board at Michigan State University and determined exempt under three study protocols. SOR MDHHS project (STUDY0002211) approved April 2019 and modified November 2020. SAMHSA MiSUPER project (STUDY00003302) approved September 2019. RHSE project (STUDY00005311) approved November 2020. Written informed consent for participation was not required for this study in accordance with the national legislation and the institutional requirements.

## Author contributions

All authors listed have made a substantial, direct, and intellectual contribution to the work and approved it for publication.

## Funding

This work was supported by State Opioid Response (SOR) funds from the Michigan Department of Health and Human Services (MDHHS); Grant No. 1H79TI082573-01 Substance Abuse Mental Health Services Administration Rural Opioid Technical Assistance; and Grant No. 2020-46100-32815 U.S. Department of Agriculture National Institute Food and Agriculture Rural Health and Safety Education (RHSE) funding program.

## Conflict of interest

The authors declare that the research was conducted in the absence of any commercial or financial relationships that could be construed as a potential conflict of interest.

## Publisher's note

All claims expressed in this article are solely those of the authors and do not necessarily represent those of their affiliated organizations, or those of the publisher, the editors and the reviewers. Any product that may be evaluated in this article, or claim that may be made by its manufacturer, is not guaranteed or endorsed by the publisher.
